# Complete mitochondrial genome of the Korean endemic earthworm *Amynthas bubonis* (Clitellata: Megascolecidae): mitogenome characterization and phylogenetic positioning

**DOI:** 10.1080/23802359.2025.2498733

**Published:** 2025-05-08

**Authors:** Jachoon Koo, Yong Hong

**Affiliations:** a Division of Science Education and Institute of Fusion Science, College of Education, Jeonbuk National University, Jeonju, Korea; b Department of Plant Medicine, College of Agriculture & Life Sciences, Jeonbuk National University, Jeonju, Republic of Korea

**Keywords:** Megascolecidae, *Amynthas bubonis*, mitochondrial genome, molecular phylogeny

## Abstract

*Amynthas bubonis* Hong & James, 2001 is an earthworm species endemic to Korea. This species is typically found in mountainous forests at low altitudes. Specimens were collected from Mt. Deogyu in South Korea, and the complete *A. bubonis* mitogenome was sequenced, assembled, and annotated. The *A. bubonic* mitogenome is a 15,095 bp circular DNA molecule with 64.85% A + T content. It contains 13 protein-coding genes, 2 ribosomal RNA genes, 22 transfer RNA genes, and 1 non-coding region (control region). Phylogenetic analysis revealed that *A. bubonis* clustered with *A. jiriensis*, *A. yunoshimensis*, and *M. hilgendorfi* in the well-supported Megascolecidae family.

## Introduction

The *Amynthas* group is the largest within the family Megascolecidae (Clitellata). Several species of the genus *Amynthas* Kinberg (1867) have been transported by humans and other organisms to various parts of the world, including the Neotropics and Nearctic regions (Gates 1972; Sims and Easton 1972). Species of the *Amynthas* group have broad ecological requirements and occupy diverse regions, soils, and vegetation types. *Amynthas* is the most abundant and diverse genus of the family Megascolecidae. *Amynthas bubonis* (Hong and James 2001) is frequently found in the forested areas of Korea.

The complete mitochondrial genome (mitogenome) of this family is available for only 27 species (Boore and Brown 1995; Wang et al. 2015; Zhang et al. 2016a, 2016b, 2016c; Hong et al. 2017; Zhang et al. 2019; Kim and Hong 2022). In the present species, another species *A. bubonis* was included, resulting in a total of 28 species. The mitogenomes of *Amynthas* species have been primarily analyzed in Asian regions, particularly China and Korea.

The *Amynthas* group is heterogeneous and encompasses various species with fasciculate and pinnate longitudinal musculature (Csuzdi & Zicsi 2003). The mitogenomic sequences of the genera *Amynthas* and Megascolecidae may provide valuable information for developing mitogenome-based phylogenies and advance our understanding of the mitogenomic evolution of Clitellata.

## Materials and methods

### Specimen collection

**Figure 1.**
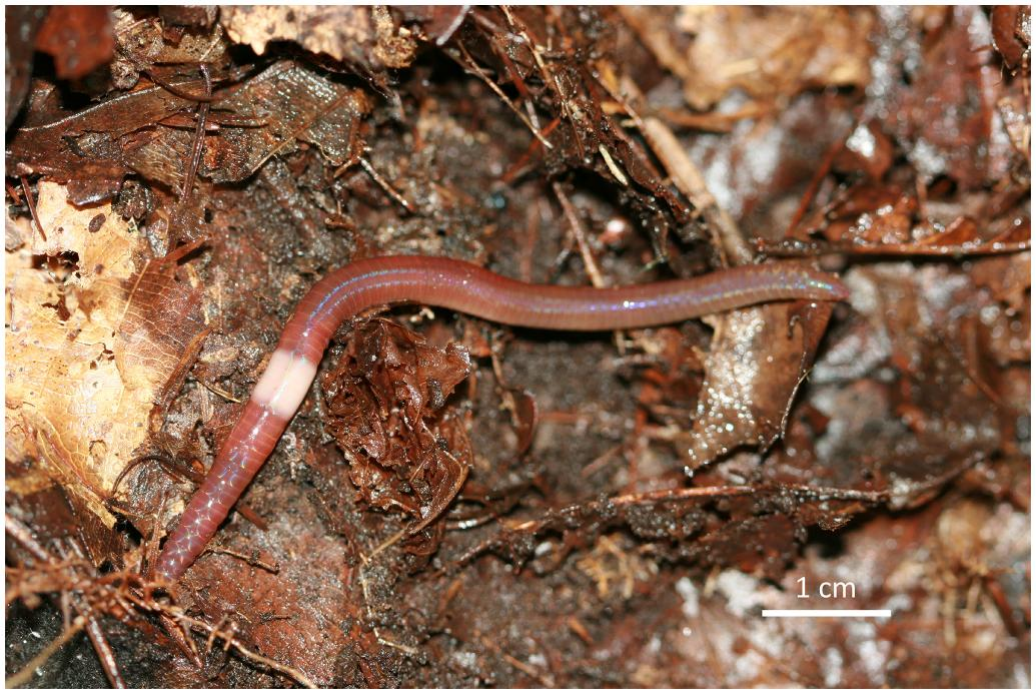
Reference image of *Amynthas bubonis* collected from Mt. Deogyu in korea. Images were captured using a Canon digital camera by Yong Hong on 9 May 2007. Scale bar: 1 cm.

*A. bubonis* (Hong and James 2001) specimens were collected from Mt. Deogyu, Jeollabuk-do, South Korea (35°86′80.11″ N, 127°81′80.83″ E; 840 m) on September 20, 2020. This material was found in the litter layers and soil of crops cultivated through manual sorting. *A. bubonis* is a brownish-red, medium-sized worm measuring 57–99 mm in length and 3.3–4.0 mm in diameter, with 64–86 segments (Figure 1; Hong and James 2001). It has two pairs of spermathecal pores located at segments 5/6–6/7, and 2–6 lateral round genital papillae.

All morphological observations were performed by external examination of whole specimens and dorsal dissection under a stereomicroscope (Zeiss KCX-160). A voucher specimen was deposited at Jeonbuk National University, Jeonju, Korea, under accession number JBNU0005 (Yong Hong, yonghong@jbnu.ac.kr).

### DNA extraction and mitogenome sequencing, assembly, and annotation

Total DNA was isolated from body segments of a single *A. bubonis* specimen using the DNeasy Blood & Tissue Kit (Qiagen, USA). The DNA was then fragmented to approximately 150 bp, and adapters were ligated to each end to produce a sequencing library using the TruSeq DNA Nano Library Kit (Illumina Inc., USA). The library was sequenced on an Illumina HiSeq-X platform (San Diego, CA, USA) to generate 39,315,704 raw reads. The Trimmomatic v 0.38 program (Bolger et al. 2014) was used to remove adapter sequences and trim bases with a base quality of less than three at the end of the read. A sliding window trimming method with a window size of four was applied to remove bases that did not meet the average quality threshold of 15. Reads shorter than 36 bp were deleted to generate 27,515,808 reads. The filtered reads were de novo assembled into a complete circular DNA sequence using SPAdes v3.13.0 (Bankevich et al. 2012). Sequencing reads were mapped to the mitogenome of *A. bubonis* (Supplementary Figure S1).

The resulting complete and circular mitogenome sequences were annotated using the MITOS web server (http://mitos2.bioinf.uni-leipzig.de/; Donath et al. 2019). To verify and refine the exact annotated gene positions, sequences were manually curated using BLAST searches in the National Center for Biotechnology Information database. A mitochondrial map was generated using the Proksee web server (https://proksee.ca/) with the relative-scale option. The filtered reads were de novo assembled into a complete circular DNA sequence using SPAdes v3.13.0 (Bankevich et al. 2012). The nucleotide composition bias was calculated using the formulas: AT-skew = [A − T]/[A + T] and GC-skew = [G − C]/[G + C].

### Phylogenetic analysis

Phylogenetic analysis was conducted using maximum likelihood (ML) with IQ-Tree and Bayesian inference (BI) with MrBayes in PhyloSuite v1.2.3 (Zhang et al. 2020), based on the nucleotide sequences of 13 mitochondrial protein-coding genes (PCGs). The mitogenome dataset included publicly available complete mitogenome sequences of 27 Megascolecidae species and one representative lumbricid species, *Lumbricus terrestris,* as an outgroup. This species is widely used in phylogenetic analysis and is the first terrestrial earthworm group to undergo complete mtDNA analysis. The nucleotide sequence of each gene was aligned using MAFFT (Katoh & Standley 2013), and gap sites were subsequently removed using trimAl (Capella-Gutiérrez et al. 2009). Analysis was performed using the program’s default settings. The aligned genes were concatenated into a single dataset to generate phylogenetic trees using the ML and BI methods.

The ML phylogeny was inferred using IQ-TREE (Nguyen et al. 2015) with the GTR+F + R5 model and 5000 ultrafast bootstraps (Minh et al. 2020). ModelFinder v2.2.0 (Kalyaanamoorthy et al. 2017) module was employed to select the BIC criterion-based best-fit partition model. For the BI analysis, phylogeny was inferred using MrBayes (Ronquist et al. 2012) under the partition model (two parallel runs, 5,000,000 generations), discarding the initial 25% of the sampled data as burn-in. We described the partition model in the following sentence as 'GTR + I + G’. PartitionFinder2 (Lanfear et al. 2017) was used to determine the optimal partitioning scheme and evolutionary models using all algorithms and the Akaike information criterion by applying the GTR+I + G model. Phylogenetic trees were visualized using FigTree 1.4.3 (Rambaut 2016), and further edited in PowerPoint.

## Results

### Complete mitochondrial genome structure of A. bubonis

**Figure 2.**
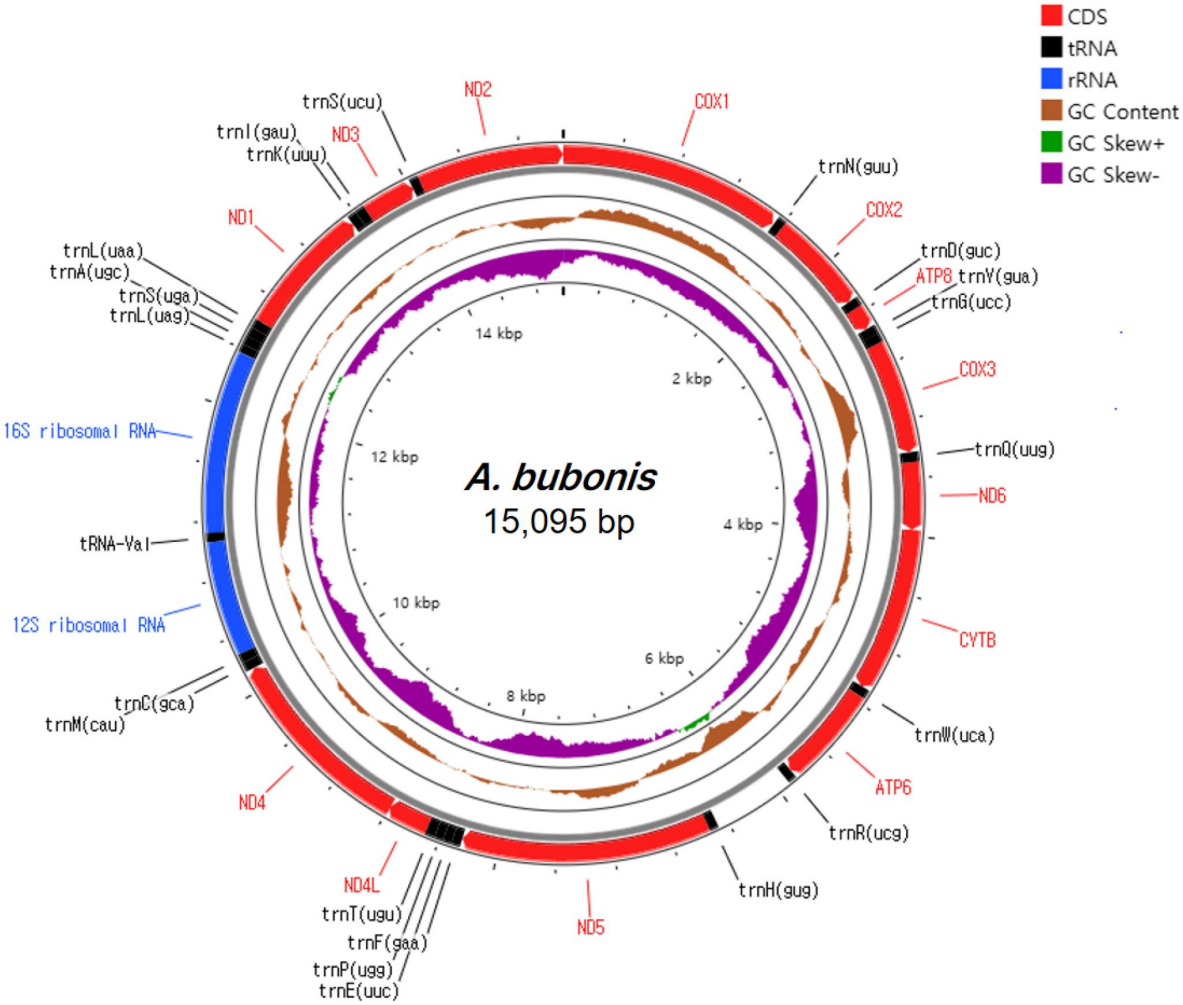
Mitochondrial map of *Amynthas bubonis*. Circular maps were generated using the proksee web server (https://proksee.ca/) with the relative scale option. Protein-coding, rRNA, and tRNA genes are shown inred, blue, and black, respectively. The GC content was plotted using a brown sliding window, as the deviation from the average GC content of the entire sequence (window size, 500 bp; step size, 1 bp). GC skew was plotted as the deviation from the average GC skew of the entire sequence, with an average value of −0.1725 (window size, 500 bp; step size, 1 bp). The innermost cycle indicates the location of the genes in the mitogenome.

The complete *A. bubonis* mitogenome is a 15,095 bp circular DNA molecule (GenBank accession no. PP949969), which contains 37 genes typical of the invertebrate mitochondrial genome (Figure 2). It includes 13 protein-coding genes (PCGs), 22 tRNAs, two rRNAs, and a control region. The gene arrangement is identical to that of megascolecid species (Boore and Brown 1995; Wang et al. 2015; Zhang et al. 2016a, 2016b, 2019). The overall nucleotide composition of the mitochondrial genome was 33% A, 33% T, 14% G, and 20% C, indicating a clear A + T bias. The A + T content of the whole mitogenome was 64.85%, similar to that found in a study on megascolecid species (61.6–67.2%). Among the PCGs, the nucleotide composition of ND6 showed the highest bias toward A + T (69%). The values of AT-Skew and GC-Skew were 0.036 and 0.162, respectively. All 13 PCGs contained ATG as the start codon. For stop codon, 13 PCGs used the conservative TAA (*COX1, ND6, CYTB, ATP6, ND4L, ND3*), TAG (*COX2, ND1*), or an incomplete stop codon T (*ATP8, COX3, ND5, ND4, ND2*).

### Genetic relationship of the family megascolecidae

**Figure 3.**
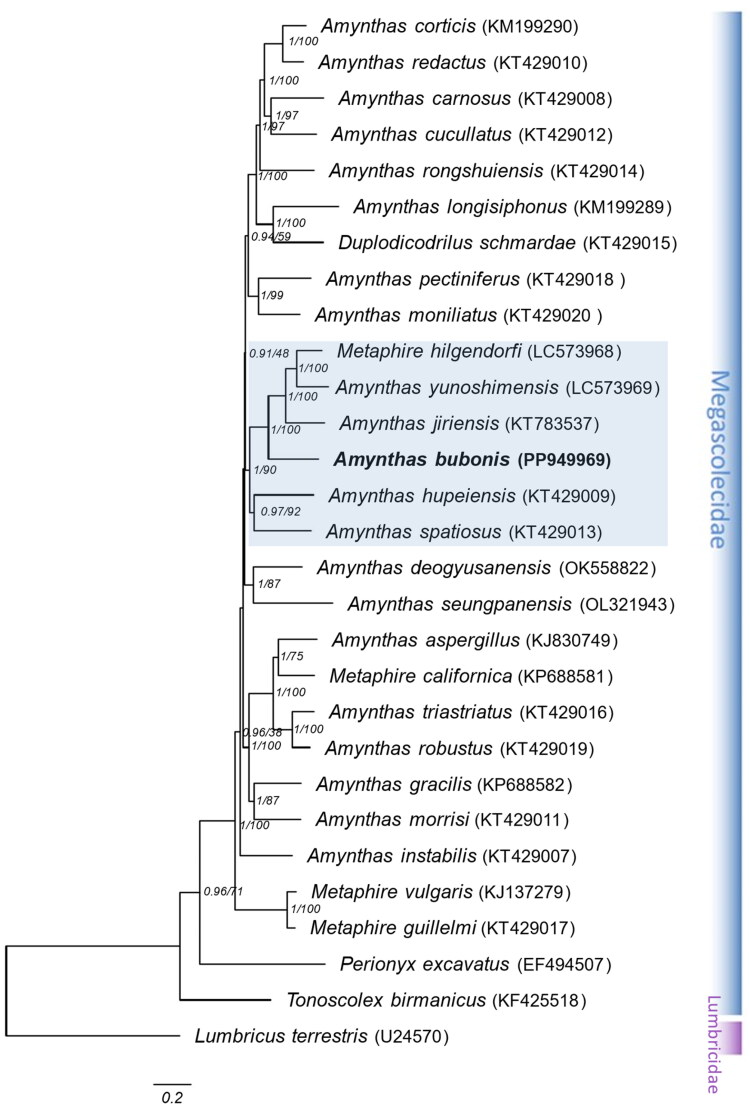
Phylogenetic relationships among 28 Megascolecidae species based on the nucleotide sequences of 13 PCGs in the mitogenome. *Lumbricus terrestris* was used as an outgroup. Phylogenetic trees were constructed using maximum likelihood (ML) and bayesian inference (BI) methods. Note that both methods yielded the same tree topology. Numbers on nodes indicate BI posterior probabilities and ML bootstrap values (%). The GenBank accession numbers were marked after the species name. The following sequences were used: *Amynthas aspergillus* (Zhang et al. 2016a), *Amynthas carnosus, Amynthas cucullatus, Amynthas hupeiensis, Amynthas instabilis, Amynthas morrisi*, *Amynthas moniliatus, Amynthas pectiniferus, Amynthas redactus, Amynthas robustus, Amynthas rongshuiensis, Amynthas spatiosus, Amynthas triastriatus, Metaphire guillelmi, Duplodicodrilus schmardae* (Zhang et al. 2016b), *Amynthas corticis, Amynthas gracilis, Amynthas longisiphonus, Metaphire californica* (Zhang et al. 2015), *Amynthas jiriensis* (Hong et al. 2017), *Amynthas seungpanensis* (Kim & Hong 2022), *Metaphire hilgendorfi, Amynthas yunoshimensis* (Seto et al. 2021), *Amynthas deogyusanensis*, *Amynthas buboniss* (this study), *Metaphire vulgaris* (Zhang et al. 2016c), *Perionyx excavatus* (unpublished), *Tonoscolex birmanicus* (Wang et al. 2015), and *Lumbricus terrestris* (Boore & Brown 1995).

*A. bubonis* phylogenetic trees prepared using both ML and BI analytical methods were highly consistent, with strong statistical support from high posterior probability and moderate bootstrap values (Figure 3). The tree showed that *A. bubonis* was sister to the clades of *M. hilgendorfi*, *A. yunoshimensis*, and *A. jiriensis*, forming a clade with high support values (1/100 for BI and bootstrap values). This clade indicates an evolutionary relationship among *Amynthas* species, with *A. bubonis* positioned within a monophyletic group that diverges from other *Amynthas* species in the Megascolecidae family.

## Discussion and conclusion

The complete mitogenome reported herein will be useful for further studies on *A. bubonis.* The branching patterns reflect the phylogenetic proximity of *A. bubonis* to other *Amynthas* species, suggesting an evolutionary relationship within this family.

Future studies should focus on analyzing additional species within this group and its sister group for the evolutionary interpretation of higher taxa among Megascolecidae and Lumbricidae. However, the information on megascolecid mitogenomes is limited. Therefore, a comprehensive analysis of the diverse genera in this group is required.

The tree analysis showed that *A. bubonis* clustered with *A. jiriensis*, *A. yunoshimensis*, and *M. hilgendorfi* within the Megascolecidae family. However, *M. hilgendorfi* remains strongly associated with the *Amynthas* group. This may suggests that *M. hilgendorfi* may belong to the genus *Amynthas* rather than *Metaphire*, as previously determined based on morphological analysis.

## Supplementary Material

Supplementary data.docx

## Data Availability

The genome sequence data supporting the findings of this study are available in the GenBank database at https://www.ncbi.nlm.nih.gov/nuccore/PP949969 (accession no. PP949969). The associated BioProject, SRA, and Bio-Sample numbers are PRJNA769829, SRS21781389, and SAMN42100821, respectively.

## References

[CIT0001] Bankevich A, Nurk S, Antipov D, Gurevich AA, Dvorkin M, Kulikov AS, Lesin VM, Nikolenko SI, Pham S, Prjibelski AD, et al. 2012. SPAdes: a new genome assembly algorithm and its applications to single-cell sequencing. J Comput Biol. 19(5):455–477. doi:10.1089/cmb.2012.0021. 22506599 PMC3342519

[CIT0002] Bolger AM, Lohse M, Usadel B. 2014. Trimmomatic: a flexible trimmer for Illumina sequence data. Bioinformatics. 30(15):2114–2120. doi:10.1093/bioinformatics/btu170. 24695404 PMC4103590

[CIT0003] Boore JL, Brown WM. 1995. Complete sequence of the mitochondrial DNA of the annelid worm *Lumbricus terrestris*. Genetics. 141(1):305–319. doi:10.1093/genetics/141.1.305. 8536978 PMC1206728

[CIT0004] Capella-Gutiérrez S, Silla-Martínez JM, Gabaldón T. 2009. trimAl: a tool for automated alignment trimming in large-scale phylogenetic analyses. Bioinformatics. 25(15):1972–1973. doi:10.1093/bioinformatics/btp348. 19505945 PMC2712344

[CIT0005] Csuzdi C, Zicsi A. 2003. Earthworms of Hungary (Annelida: Oligochaeta, Lumbricidae). Pedozoologica Hung. 1:1–271.

[CIT0006] Donath A, Jühling F, Al-Arab M, Bernhart SH, Reinhardt F, Stadler PF, Middendorf M, Bernt M. 2019. Improved annotation of protein-coding genes boundaries in metazoan mitochondrial genomes. Nucleic Acids Res. 47(20):10543–10552. doi:10.1093/nar/gkz833. 31584075 PMC6847864

[CIT0007] Gates GE. 1972. Burmese earthworms. An introduction to the systematics and biology ofmegadrile oligochaetes with special reference to Southeast Asia. Trans Am Philos Soc. 62(7):1–326. doi:10.2307/1006214.

[CIT0009] Hong Y, James SW. 2001. New species of Korean *Amynthas* Kinberg, 1867 (Oligochaeta, Megascolecidae) with two pairs of spermathecae. Rev Suisse Zool. 108:65–93. doi:10.5962/bhl.part.79621.

[CIT0010] Hong Y, Kim MJ, Wang AR, Kim IK. 2017. Complete mitochondrial genome of the earthworm, *Amynthas jiriensis* (Clitellata: megascolecidae). Mitochondrial DNA A DNA Mapp Seq Anal. 28(2):163–164. doi:10.3109/19401736.2015.1115491. 26709745

[CIT0011] Kalyaanamoorthy S, Minh BQ, Wong TK, von Haeseler A, Jermiin LS. 2017. ModelFinder: fast model selection for accurate phylogenetic estimates. Nat Methods. 14(6):587–589. doi:10.1038/nmeth.4285. 28481363 PMC5453245

[CIT0012] Katoh K, Standley DM. 2013. MAFFT multiple sequence alignment software version 7: improvements in performance and usability. Mol Biol Evol. 30(4):772–780. doi:10.1093/molbev/mst010. 23329690 PMC3603318

[CIT0013] Kim MJ, Hong Y. 2022. Complete mitochondrial genome of the earthworm *Amynthas seungpanensis* (Clitellata: megascolecidae). Mitochondrial DNA B Resour. 7(6):989–991. doi:10.1080/23802359.2022.2080604. 35712539 PMC9196842

[CIT0014] Lanfear R, Frandsen PB, Wright AM, Senfeld T, Calcott B. 2017. PartitionFinder 2: new methods for selecting partitioned models of evolution for molecular and morphological phylogenetic analyses. Mol Biol Evol. 34(3):772–773. doi:10.1093/molbev/msw260. 28013191

[CIT0015] Minh BQ, Hahn MW, Lanfear R. 2020. New methods to calculate concordance factors for phylogenomic datasets. Mol Biol Evol. 37(9):2727–2733. doi:10.1093/molbev/msaa106. 32365179 PMC7475031

[CIT0016] Nguyen LT, Schmidt HA, von Haeseler A, Minh BQ. 2015. IQ-TREE: a fasand effective stochastic algorithm for estimating maximum-likelihood phylogenies. Mol Biol Evol. 32(1):268–274. doi:10.1093/molbev/msu300. 25371430 PMC4271533

[CIT0017] Rambaut A. 2016. FigTree v1.4.3 [Computer software]. http://tree.bio.ed.ac.uk/software/figtree/.

[CIT0018] Ronquist F, Teslenko M, van der Mark P, Ayres DL, Darling A, Höhna S, Larget B, Liu L, Suchard MA, Huelsenbeck JP. 2012. MrBayes 3.2: efficient Bayesian phylogenetic inference and model choice across a large model space. Syst Biol. 61(3):539–542. doi:10.1093/sysbio/sys029. 22357727 PMC3329765

[CIT0019] Seto A, Endo H, Minamiya Y, Matsuda M. 2021. The complete mitochondrial genome sequences of Japanese earthworms *Metaphire hilgendorfi* and *Amynthas yunoshimensis* (Clitellata: megascolecidae). Mitochondrial DNA B Resour. 6(3):965–967. doi:10.1080/23802359.2020.1830728. 33796700 PMC7995826

[CIT0020] Sims RW, Easton EG. 1972. A numerical revision of the earthworm genus *Pheretima* auct. (Megascolecidae: oligochaeta) with the recognition of new genera and an appendix on the earthworms collected by the Royal Society North Borneo Expedition. Biol J Linnean Soc. 4(3):169–268. doi:10.1111/j.1095-8312.1972.tb00694.x.

[CIT0021] Wang AR, Hong Y, Win TM, Kim I. 2015. Complete mitochondrial genome of the Burmese giant earthworm, *Tonoscolex birmanicus* (Clitellata: megascolecidae). Mitochondrial DNA. 26(3):467–468. doi:10.3109/19401736.2013.830300. 24047177

[CIT0022] Zhang D, Gao FL, Jakovlić I, Zou H, Zhang J, Li WX, Wang GT. 2020. PhyloSuite: an integrated and scalable desktop platform for streamlined molecular sequence data management and evolutionary phylogenetics studies. Mol Ecol Resour. 20(1):348–355. doi:10.1111/1755-0998.13096. 31599058

[CIT0023] Zhang L, Jiang J, Dong Y, Qiu J. 2015. Complete mitochondrial genome of four pheretimoid earthworms (Clitellata: oligochaeta) and their phylogenetic reconstruction. Gene. 574(2):308–316. doi:10.1016/j.gene.2015.08.020. 26291739

[CIT0024] Zhang L, Jiang J, Dong Y, Qiu J. 2016a. Complete mitochondrial genome of an *Amynthas* earthworm*, Amynthas aspergillus* (Oligochaeta: megascolecidae). Mitochondrial DNA A DNA Mapp Seq Anal. 27(3):1876–1877. doi:10.3109/19401736.2014.971267. 25329289

[CIT0025] Zhang L, Sechi P, Yuan M, Jiang J, Dong Y, Qiu J. 2016b. Fifteen new earthworm mitogenomes shed new light on phylogeny within the *Pheretima* complex. Sci Rep. 6(1):20096. doi:10.1038/srep20096. 26833286 PMC4735579

[CIT0026] Zhang L, Jiang J, Dong Y, Qiu J. 2016c. Complete mitochondrial genome of a Pheretimoid earthworm *Metaphire vulgaris* (Oligochaeta: megascolecidae). Mitochondrial DNA A DNA Mapp Seq Anal. 27(1):297–298. doi:10.3109/19401736.2014.892085. 24617491

[CIT0027] Zhang Q, Liu H, Zhang Y, Ruan H. 2019. The complete mitochondrial genome of *Lumbricus rubellus* (Oligochaeta, Lumbricidae) and its phylogenetic analysis. Mitochondrial DNA B Resour. 4(2):2677–2678. doi:10.1080/23802359.2019.1644242. 33365679 PMC7706552

