# Supplementary material for: Complete mitochondrial genome of the Korean endemic earthworm Amynthas bubonis (Clitellata: Megascolecidae): mitogenome characterization and phylogenetic positioning
Source: Mitochondrial DNA B Resour. 2025 May 8;10(6):437–41. doi: 10.1080/23802359.2025.2498733 (PMC12064099; doi:10.1080/23802359.2025.2498733)
Supplement: Supplementary data.docx [file TMDN_A_2498733_SM7279.docx]

**Supplementary data**

**Complete mitochondrial genome of the earthworm *Amynthas bubonis* (Clitellata: Megascolecidae): Mitogenome characterization and phylogenetic positioning**

Jachoon Koo^1^, and Yong Hong^2^

^1^Division of Science Education and Institute of Fusion Science, College of Education, Jeonbuk National University, Jeonju 54896, Korea; [jkoo@jbnu.ac.kr](mailto:jkoo@jbnu.ac.kr) https://orcid.org/ 0000-0002-3559-326X

^2^Department of Agricultural Biology, College of Agriculture & Life Sciences, Jeonbuk National University, Jeonju 54896, Republic of Korea; yonghong@jbnu.ac.kr <https://orcid.org/0000-0002--8093-9717>

^2^ Correspondence:

Yong Hong

Tel: +82-63-270-2529, Fax: +82-63-270-2531, e-mail: yonghong@jbnu.ac.kr


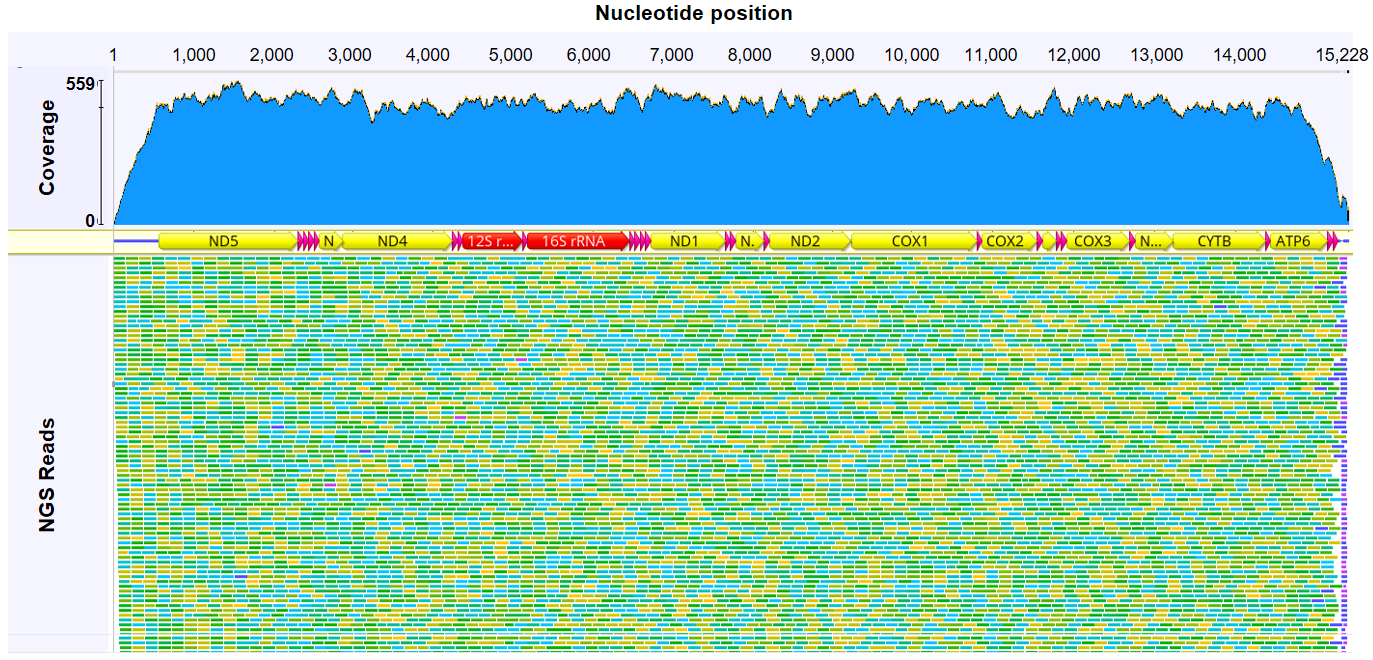


Supplementary Figure S1. Coverage plot of mitogenome of *Amynthas bubonis*. Among 166,854 reads, the 97,533 reads were mapped to an assembly result (100% of coverage, 147.94 of average mapping depth) using Bowtie2 in Genius prime (ver. 2023.0.2).
